# A ‘ghost’ after transvenous intracardiac lead extraction

**DOI:** 10.1007/s12471-022-01754-z

**Published:** 2023-01-10

**Authors:** V. Neto, J. Santos, N. Craveiro, L. Santos, M. Correia

**Affiliations:** Department of Cardiology, Tondela-Viseu Hospital Centre, Viseu, Portugal

A 49-year-old man with a history of familial amyloidotic polyneuropathy was admitted to the hospital because of device-related endocarditis. He had undergone a liver transplant and placement of a single-chamber pacemaker 19 years earlier. After transvenous right ventricular lead extraction (LE), transthoracic echocardiography revealed a hyperechogenic, filiform, anomalous mass of 8 × 5 mm (Fig. 1, and see Videos 1 and 2 in Electronic Supplementary Material). The mass was located along the removed lead’s intracardiac route; one end of the mass was attached to the right ventricular wall and the other end was located below the tricuspid valve and had a very mobile tip. An interventional report and an X‑ray confirmed complete LE. The mass was interpreted as a thick, fibrous, tubular encasement of the lead that persisted after extraction, also known as ‘ghost’. A conservative approach was followed. Blood cultures were persistently negative, and the patient remained asymptomatic during subsequent follow-up.

**Fig. 1:**
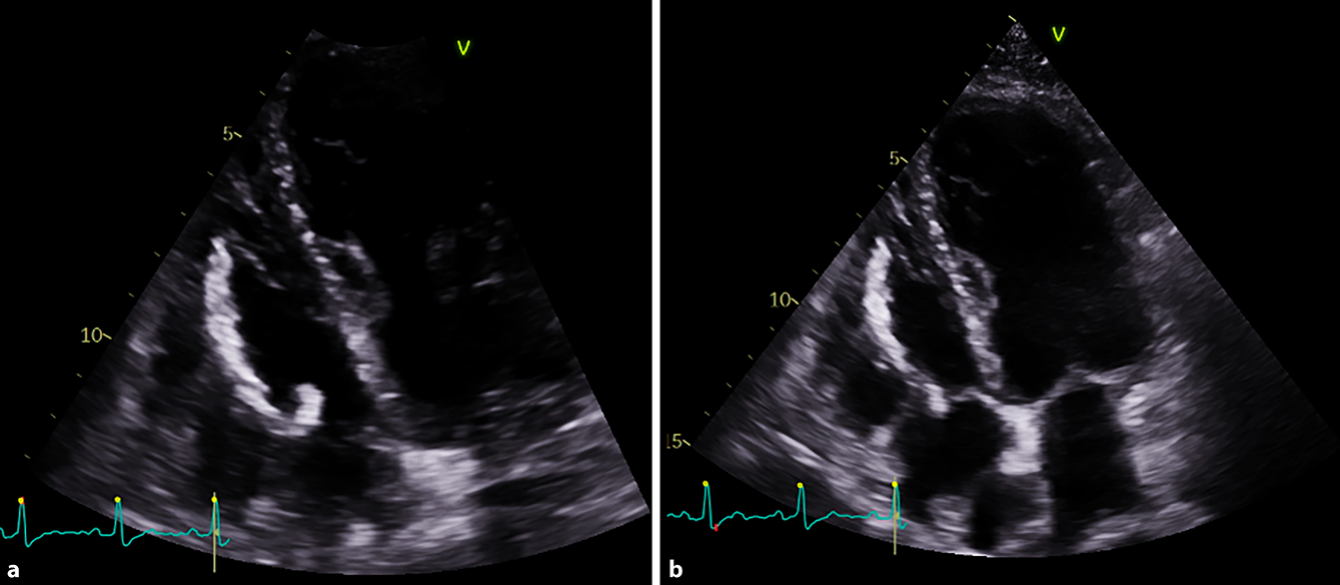
Transthoracic echocardiography. **a** Four-chamber right-ventricle focused view showing hyperechogenic, filiform, anomalous mass (8 × 5 mm), with mobile tip curving inside tricuspid valve. **b** Four-chamber view showing attachments of mass to right ventricular wall and below tricuspid valve

The presence of ‘ghosts’ is associated with a poor prognosis after LE, but the best approach for patients with this finding remains unclear. To prevent complications such as recurrent infective endocarditis or embolic events, close follow-up is needed [1].

## Supplementary Information

**Video 1** Transthoracic echocardiogram after lead extraction

**Video 2** Three-dimensional evaluation of mass in transthoracic echocardiogram after lead extraction
